# A phase I/II trial of sorafenib and infliximab in advanced renal cell carcinoma

**DOI:** 10.1038/sj.bjc.6605889

**Published:** 2010-09-14

**Authors:** J M G Larkin, T R Ferguson, L M Pickering, K Edmonds, M G James, K Thomas, U Banerji, B Berns, C de Boer, M E Gore

**Affiliations:** 1 Department of Renal Oncology, The Royal Marsden Hospital NHS Foundation Trust, Fulham Road, London, SW36JJ, UK; 2 Ortho Biotech Oncology Research & Development, 50-100 Holmers Farm Way, High Wycombe, Buckinghamshire HP12 4DP, UK; 3 Division of Centocor B.V., Ortho Biotech Oncology Research & Development, Einsteinweg 92 2333 CD, Leiden, The Netherlands

**Keywords:** renal cell carcinoma, sorafenib, infliximab

## Abstract

BACKGROUND: There is clinical evidence to suggest that tumour necrosis factor-*α* (TNF-*α*) may be a therapeutic target in renal cell carcinoma (RCC). Multi-targeted kinase inhibitors, such as sorafenib and sunitinib, have become standard of care in advanced RCC. The anti-TNF-*α* monoclonal antibody infliximab and sorafenib have differing cellular mechanisms of action. We conducted a phase I/II trial to determine the safety and efficacy of infliximab in combination with sorafenib in patients with advanced RCC.

METHODS: Eligible patients were systemic treatment-naive or had received previous cytokine therapy only. Sorafenib and infliximab were administered according to standard schedules. The study had two phases: in phase I, the safety and toxicity of the combination of full-dose sorafenib and two dose levels of infliximab were evaluated in three and three patients, respectively, and in phase II, further safety, toxicity and efficacy data were collected in an expanded patient population.

RESULTS: Acceptable safety was reported for the first three patients (infliximab 5 mg kg^−1^) in phase 1. Sorafenib 400 mg twice daily and infliximab 10 mg kg^−1^ were administered to a total of 13 patients (three in phase 1 and 10 in phase 2). Adverse events included grade 3 hand–foot syndrome (31%), rash (25%), fatigue (19%) and infection (19%). Although manageable, toxicity resulted in 75% of the patients requiring at least one dose reduction and 81% requiring at least one dose delay of sorafenib. Four patients were progression-free at 6 months (PFS_6_ 31%); median PFS and overall survival were 6 and 14 months, respectively.

CONCLUSION: Sorafenib and infliximab can be administered in combination, but a significant increase in the numbers of adverse events requiring dose adjustments of sorafenib was observed. There was no evidence of increased efficacy compared with sorafenib alone in advanced RCC. The combination of sorafenib and infliximab does not warrant further evaluation in patients with advanced RCC.

The treatment of metastatic renal cell carcinoma (RCC) has evolved rapidly since 2006 with the clinical development of targeted agents, such as sorafenib and sunitinib. Sorafenib is an inhibitor of multiple tyrosine kinases, including vascular endothelial growth factor (VEGF) and platelet derived growth factor (PDGF) receptors; the recommended dose is 400 mg twice daily; dose-limiting toxicities include diarrhoea, fatigue and skin toxicity (Strumberg *et al*, 2005). The activity of sorafenib in patients with advanced RCC has been demonstrated in the target phase III placebo-controlled trial in cytokine-pretreated patients (Escudier *et al*, 2007) that led to registration of the drug in this indication. A recently published randomised phase II study in previously untreated patients, however, did not show a progression-free survival (PFS) advantage for sorafenib in comparison with interferon-*α* (5.7 *vs* 5.6 months, respectively) (Escudier *et al*, 2009).

Infliximab is a chimeric human–mouse monoclonal antibody to tumour necrosis factor-*α* (TNF-*α*), a pro-inflammatory cytokine. Infliximab prevents TNF-*α* binding to receptors, thereby neutralising its activity. *In-vitro* models suggest that this can induce cell death by complement-mediated lysis through the interaction with membrane-bound TNF-*α* (Scallon *et al*, 1995). Although animal studies using TNF-*α* in high dose can induce significant anti-cancer effects, (Locksley *et al*, 2001; Balkwill, 2002, 2006), lower levels of TNF-*α* may be involved in cancer promotion, tumour growth and metastasis, either directly or by a network of cytokines, chemokines and matrix metalloproteinases (Moore *et al*, 1999; Locksley *et al*, 2001; Balkwill, 2006). TNF-*α* also has a role in cancer cachexia and fatigue and is a putative autocrine and paracrine growth factor in RCC (Mizutani *et al*, 1994; Balkwill, 2006).

Infliximab is licensed for use in inflammatory diseases at doses of 3–10 mg kg^−1^. Large randomised trials have documented the safety of infliximab; the most common adverse events include injection site or infusion reactions, development of anti-nuclear antibodies and infection (Lichtenstein *et al*, 2009). In 2007, we published the results of two sequential phase II trials documenting the activity of the anti-TNF-*α* antibody infliximab at dose levels of 5 and 10 mg kg^−1^ in patients with metastatic RCC previously treated with cytokine therapy (Harrison *et al*, 2006). Of the 37 patients treated, three patients achieved a partial response and 46% achieved clinical benefit (partial response or stable disease >3 months), which was considered clinically significant given that all patients had documented disease progression at the time of study entry. Infliximab was well tolerated. A dose of 10 mg kg^−1^ was recommended for further evaluation in the treatment of advanced RCC. Although outcomes for patients with advanced RCC have improved significantly in recent years with the development of novel targeted systemic therapies, almost all patients develop resistance to treatment and cure is rarely seen (Miller and Larkin 2009). Consequently there remains a need to induce longer lasting remissions, to overcome resistance and to improve survival. A potential approach to this is to combine active agents, and given that sorafenib and infliximab have different mechanisms of action and have non-overlapping toxicity profiles, we carried out a phase I/II study to explore the safety and efficacy of this combination at the full single agent doses of both drugs.

## Patients and methods

### Study objectives and patient selection

Multi-targeted kinase inhibitors and anti-TNF-*α* therapy have not previously been combined in humans, so the study was conducted in two parts: phase I and phase II. The objective of phase I was to assess the safety and toxicity of the combination of two dose levels of infliximab and full-dose sorafenib. The objective of phase II was to carry out a preliminary assessment of the efficacy of the combination and to gather further safety and toxicity data. Study inclusion criteria included: histologically proven metastatic RCC; measurable disease according to RECIST 1.0 (Therasse *et al*, 2000); either systemic treatment-naive or have progressed after immunotherapy; Eastern Cooperative Oncology Group performance status of 0 or 1; adequate bone marrow, liver and renal function.

### Study design and statistical methods

This trial was conducted at The Royal Marsden Hospital NHS Foundation Trust and recruited between June 2007 and February 2009. Approval for the study was obtained from the Local Research Ethics Committee and Regulatory Authority. All patients provided written informed consent before study entry.

Phase I was carried out according to a standard three and three design, respecyively: the first cohort of three patients was treated at full-dose sorafenib 400 mg twice per day (bd) orally and infliximab 5 mg kg^−1^ intravenously. In the absence of significant safety and toxicity problems in the first cohort, the dose of infliximab was to be escalated to the full dose of 10 mg kg^−1^ in combination with sorafenib 400 mg bd in the second cohort of three patients. Sorafenib was started on day 0 with standard dose reductions and interruptions for toxicity as necessary. Infliximab was administered at weeks 1, 3 and 7 and then every 4 weeks. In the event of dose delays due to toxicity from one agent, administration of the other agent continued in the absence of contraindications.

Phase II was carried according to a Simon two-stage design; the primary end point was the proportion of patients who were progression-free at 6 months (PFS_6_). Secondary endpoints included toxicity, overall survival and response rate at 12 weeks. It was assumed that if the proportion of patients alive and PFS_6_ was <47% then the combination of sorafenib and infliximab did not demonstrate additive activity. According to Simon, the detection of a PFS_6_ rate of ⩾47% with a probability of a type I error of 5% and a type II error of 20% required the enrolment of 18 patients in stage one. Of the 18 patients, 10 or more were required to be PFS_6_ to proceed to stage two. To minimise the chance of patients being subjected to a potentially toxic treatment, the efficacy analysis population consisted of all patients treated in phase II and all patients in phase I treated at the same doses of sorafenib and infliximab.

### Response and toxicity assessments

Response to therapy was assessed using the Response Evaluation Criteria in Solid Tumours (Therasse *et al*, 2000), with computed tomographic scans at baseline and every 12 weeks following the initiation of treatment. Adverse events were graded using the National Cancer Institute Common Toxicity Criteria version 3.0.

## Results

A total of 16 patients were recruited; six into the phase I and 10 into the phase II parts of the study. Baseline patient characteristics are shown in Table 1. Patients had an average age of 57 (range, 35–72), were predominantly male (13 of 16 patients) and most (12 of 16 patients) were in the low-risk Memorial Sloan-Kettering Cancer Centre prognostic risk category (Motzer *et al*, 2002). Cytokine therapy had been administered previously to five patients, and most patients (14 of 16 patients) had undergone previous nephrectomy. All patients except two had clear cell histology and all patients except one had documented disease progression at the time of study entry. The patient who did not have evidence of radiological progression at the time of study entry had multiple lung metastases requiring first line systemic treatment.

In phase I, no unexpected toxicities were seen in the three patients treated with sorafenib 400 mg bd and infliximab 5 mg kg^−1^; the next cohort of three patients was, therefore, dosed at sorafenib 400 mg bd and infliximab 10 mg kg^−1^. One patient at this dose level developed grade three fatigue and grade 3 rash and the other two experienced grade 3 hand–foot–skin reaction. All of these toxicities were considered attributable to sorafenib rather than the combination of agents, and as a consequence these doses were selected for further evaluation in the phase II part of the study given our desire in the expansion to further evaluate the efficacy and toxicity of infliximab and sorafenib at the recommended therapeutic doses of both agents in RCC.

**Figure 1:**
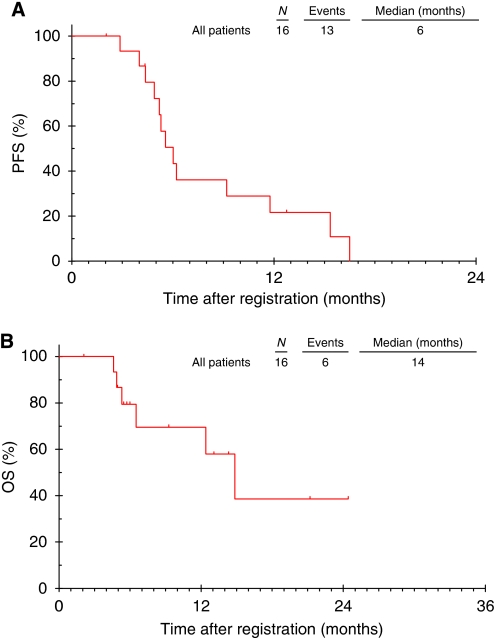
Kaplan–Meier plots of (**A**) progression-free survival (PFS) and (**B**) overall survival (OS).

For the analysis of efficacy, data from the three patients dosed at sorafenib 400 mg bd and infliximab 10 mg kg^−1^ in phase I were combined with data from the 10 patients treated in phase II. Four of the 13 patients were PFS_6_ (31%); an interim efficacy analysis was performed and the trial was closed at this point because the pre-specified target of ⩾10 of 18 patients achieving PFS_6_ in stage one was not attainable. One patient achieved a partial response, two patients had progressive disease and the remaining 10 patients had stable disease as their best response to treatment. The Kaplan–Meier median PFS (Figure 1A) for all 16 patients from phase 1 and 2 was 6 months (95% confidence interval 4.8–7.2 months) and the Kaplan–Meier median overall survival (Figure 1B) for all patients was 14 months (95% confidence interval 10–19 months).

One patient in phase I and three patients in phase II stopped study treatment because of adverse reactions and two patients remained on study treatment at the time of the last follow-up. Of the four patients who stopped because of the adverse reactions, two did so because of the serious infections, one because of allergic reaction and one because of the development of multiple toxicities that included hand–foot syndrome, fatigue and mucositis. All other patients discontinued treatment because of the progressive disease. Adverse events for all patients are summarised in Table 2. There were no grade 4 adverse events and no treatment-related deaths. Grade 3 adverse events were experienced by 13 of 16 patients and the three remaining patients all reported grade 2 adverse events. The most common grade 3 adverse events were hand–foot syndrome (31%), rash (25%), fatigue (19%) and infection (19%). The most frequent adverse events of any grade were rash (88%), lymphopaenia (81%), diarrhoea (81%), alopecia (75%) and hand–foot syndrome (75%). Serious haematological toxicity was uncommon. Serious infection occurred in two patients; both developed infections within primary renal tumours and the surrounding renal parenchyma with associated abscess formation. Allergic reactions were reported in two patients; one to infliximab and one to sorafenib. Of 16 patients, 5 experienced dose delays of infliximab and 13 had delays of sorafenib. A total of 12 patients had a reduction in sorafenib dose to once daily and, of these, three had a further reduction to once every 2 days. None of the patients had a reduction in infliximab dose. We did not observe a reduction of potentially TNF-*α* mediated constitutional symptoms, such as anorexia or lethargy.

## Discussion

We investigated the safety and efficacy of combining sorafenib with infliximab for the treatment of advanced RCC. To our knowledge, this is the first report of the combination of a multi-targeted kinase inhibitor with anti-TNF-*α* therapy in humans.

We evaluated a dose of sorafenib 400 mg twice daily and infliximab 10 mg kg^−1^ every 4 weeks. Only four of 13 patients (31%) treated with this combination were free from progression 6 months after commencing treatment; this is lower than would be predicted with sorafenib alone. We enrolled a mixture of patients who were naive to systemic treatment and others who had progressed after immunotherapy. The activity of sorafenib in these settings may be regarded similar. In a randomised phase II trial of 189 previously untreated patients, the median PFS on sorafenib was 5.7 months with an estimated PFS_6_ 47% (Escudier *et al*, 2009) and in a phase III trial of 903 previously treated patients (83% with cytokines), the median PFS on sorafenib was 5.5 months and estimated PFS_6_ 43% (Escudier *et al*, 2007).

The lack of efficacy for the combination of sorafenib and infliximab cannot be explained by baseline patient factors given that the study population consisted mainly of patients of low or intermediate Memorial Sloan-Kettering Cancer Centre risk. Two patients had not undergone previous nephrectomy. In this study there was a considerably higher rate of sorafenib dose reductions, delays and treatment discontinuation in comparison with rates reported in the literature for sorafenib alone (Escudier *et al*, 2007, 2009). We observed a discontinuation rate because of the adverse reactions of 25%, a dose reduction rate for sorafenib of 75% and a dose interruption rate for sorafenib of 81%. In comparison, in the target study (Escudier *et al*, 2007) discontinuation and dose reduction rates were 10 and 13%, respectively, with dose interruptions in 21%. It is possible that the lower dose intensity of sorafenib treatment in our study due to these dose adjustments contributed to reduced efficacy, particularly as a dose-dependent increase in efficacy of sorafenib has been reported (Amato *et al*, 2007; Escudier *et al*, 2009).

It is unclear whether the relatively lower dose intensity of sorafenib can be attributed to increased toxicity resulting from the concurrent administration of infliximab, or whether it is a chance finding given the small sample size. The aim of this study was to deliver therapeutic single agent doses of both agents in combination as sub-therapeutic doses might lead to rejection of a potentially efficacious combination. The toxicity seen in the phase II part of this study was higher than that reported for sorafenib as a single agent in two pivotal trials that included 189 and 903 patients, respectively (Escudier *et al*, 2007, 2009). The following adverse reactions were more frequent in our study compared with those reported in patients treated with sorafenib in the target study (Escudier *et al*, 2007); rash (88 *vs* 40%), diarrhoea (81 *vs* 43%), alopecia (75 *vs* 27%), hand–foot syndrome (75 *vs* 30%), anaemia (69 *vs* 8%), fatigue/lethargy (62 *vs* 37%), dyspnoea (44 *vs* 14%), anorexia (31 *vs* 16%), nausea (37 *vs* 23%) and hypertension (25 *vs*17%). Although most of the increased toxicity in our study was due to an increase in grade 1 or grade 2 events, grade 3 or 4 toxicity, including rash (25 *vs* 1%), hand–foot syndrome (31 *vs* 6%) and lethargy (19 *vs* 5%) was also frequently observed. It is of note that, two of our patients developed serious infections with abscess formation in primary renal tumours/surrounding renal parenchyma. It is possible that the use of infliximab contributed to this given that immunosupression is a known side effect of this agent.

This study suggests that the combination of sorafenib and infliximab at full single dose levels should not be further evaluated in patients with advanced RCC. However, the putative anti-tumour activity of infliximab that has been previously demonstrated in advanced RCC (Harrison *et al*, 2006) warrants further investigation and combination with alternative agents or in subgroups of patients should be considered.

## Figures and Tables

**Table 1:** Baseline patient characteristics

**Characteristic**	**Number (%)**
Age/median (range)57 (35–72) years	
	
*Performance status*	
0	7 (44)
1	9 (56)
	
*Gender*	
Male	13 (81)
Female	3 (19)
	
*Histology*	
Clear cell	14 (88)
Clear cell/sarcomatoid	1 (6)
Clear cell/papillary	1 (6)
	
*Grade*	
G2	4 (25)
G3	7 (44)
G4	4 (25)
GX	1 (6)
	
*Disease status at study entry*	
Progressive disease	15 (94)
Stable disease	1 (6)
	
*MSKCC risk group*	
High	1 (6)
Intermediate	3 (19)
Low	12 (75)
	
*Previous nephrectomy*	
Yes	14 (88)
No	2 (12)
	
*Previous systemic treatment*	
Cytokine	5 (31)
Nil	11 (69)

Abbreviation: MSKCC=Memorial Sloan-Kettering Cancer Centre.

**Table 2:** Treatment-related adverse events (worst grades, all patients)

	**Grade 1**		**Grade 2**		**Grade 3**		**Grade 4**		**Any Grade**	
**Adverse event**	***n***	(%)	***n***	(%)	***n***	(%)	***n***	(%)	***n***	(%)
Rash	5	31	5	31	4	25	—	—	14	88
Lymphopaenia	12	75	—	—	1	6	—	—	13	81
Diarrhoea	10	62	2	12	1	6	—	—	13	81
Alopecia	10	62	2	12	—	—	—	—	12	75
Hand–foot reaction	3	19	4	12	5	31	—	—	12	75
Anaemia	8	50	2	12	1	6	—	—	11	69
Fatigue/lethargy	4	25	3	19	3	19	—	—	10	62
Stomatitis/mucositis	6	37	3	19	—	—	—	—	9	56
Infection	3	19	2	12	2	12	—	—	7	44
Dyspnoea	6	37	1	6	—	—	—	—	7	44
Nausea/vomiting	4	25	1	6	1	6	—	—	6	37
Flushing	5	31	—	—	1	6	—	—	6	37
Anorexia	4	25	1	6	—	—	—	—	5	31
Constipation	3	19	1	6	—	—	—	—	4	25
Leucopaenia	2	12	1	6	1	6	—	—	4	25
Thrombocytopaenia	4	25	—	—	—	—	—	—	4	25
Hypertension	—	—	3	19	1	6	—	—	4	25
Neutropaenia	1	6	1	6	—	—	—	—	2	12
Other	7	44	2	12	7	44	—	—	16	100
